# Supplementary figures and images for: Phosphorylation/dephosphorylation response to light stimuli of Symbiodinium proteins: specific light-induced dephosphorylation of an HSP-like 75 kDa protein from S. microadriaticum
Source: PeerJ. 2019 Aug 12;7:e7406. doi: 10.7717/peerj.7406 (PMC6694782; doi:10.7717/peerj.7406)

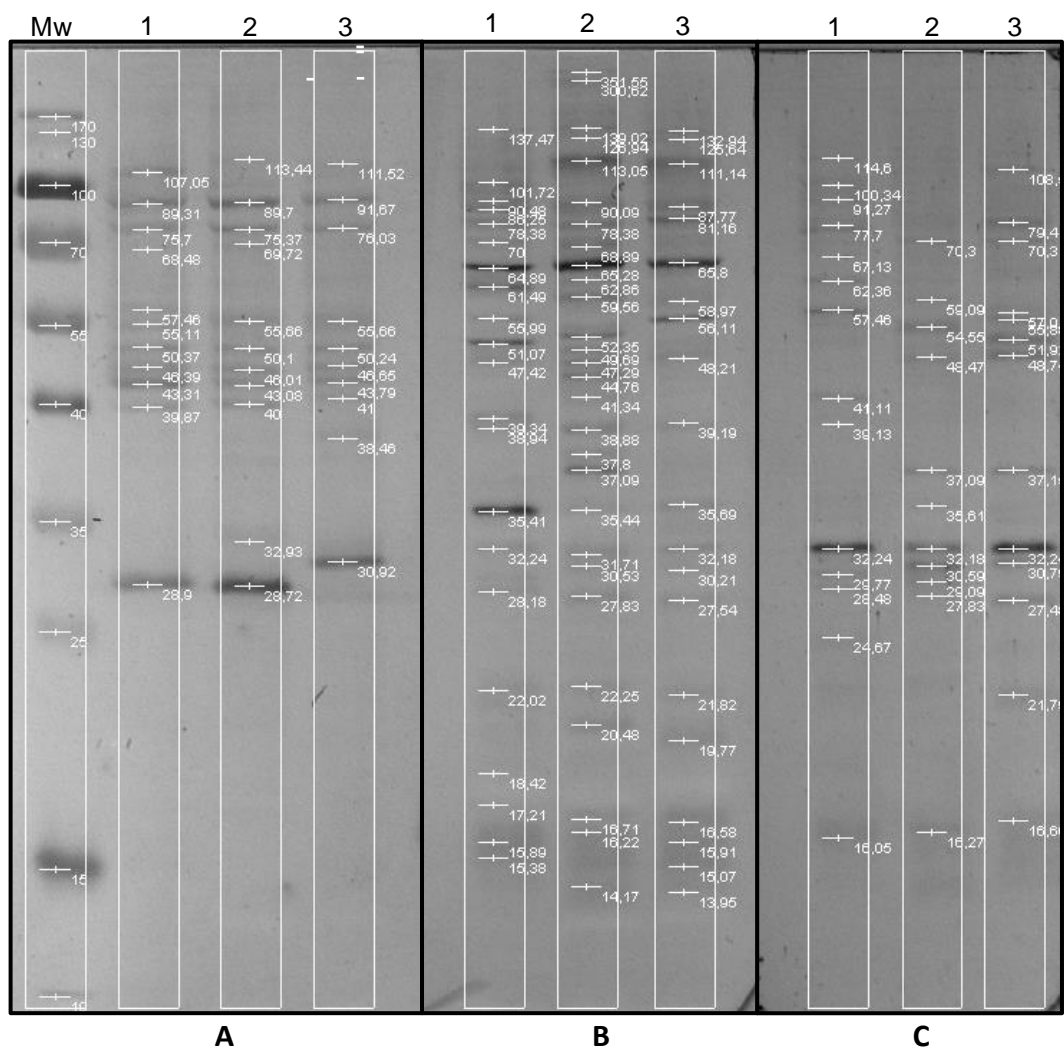

Supplement: Figure S1 — Relative molecular weights of the phospho proteins shown in Figure 1 and corresponding to the immunodetection by western blot with anti-pThr (A), -pSer (B), and -pTyr (C) antibodies in protein extracts of Symbiodinium KB8 (lanes 1), Symbiodinium Mf11 (lanes 2) and Symbiodinium kawagutii (lanes 3). [file peerj-07-7406-s001.pdf]

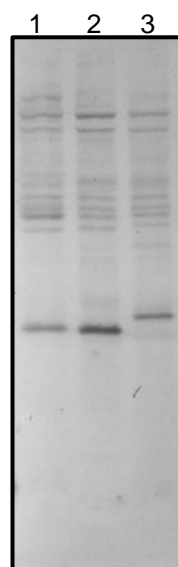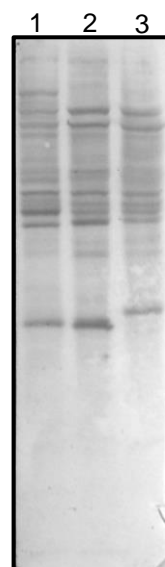

Supplement: Figure S2 — Extracts after dark incubation for 12 h from Symbiodinium KB8 (lanes 1), Symbiodinium Mf11 (lanes 2) and Symbiodinium kawagutii (lanes 3), were analyzed by western blot with anti-pThr antibodies using incubations, washes and development in either PBS-T (A) or TBS-T (B). [file peerj-07-7406-s002.pdf]

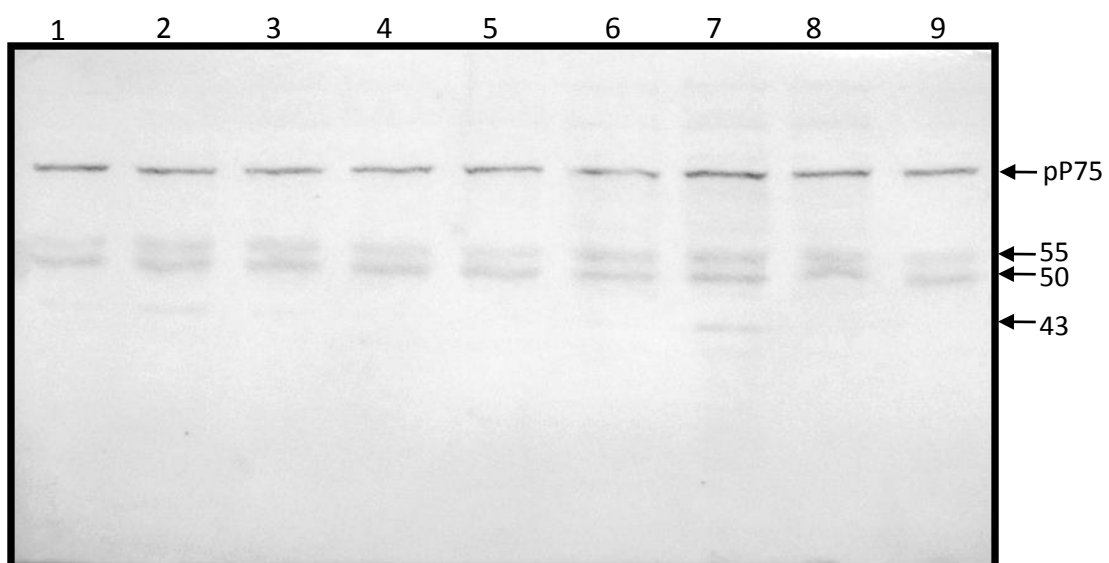

Supplement: Figure S3 — Extracts of proteins from cells without stimulation (control, lane 1) or added with: 0.1% (w/v) hydrolyzed casein (lane 2), 10 mM glycine (lane 3), 10 mM arginine (lane 4), 10 mM glutamic acid (lane 5), 100 µg/ml of RGD (lane 6) or RAD (lane 7) tripeptide, 15 mM EGTA (lane 8), or 20 mM CaCl2 (lane 9). The arrows to the right indicate the molecular weights (kDa) of the proteins immunodetected with the anti-pThr antibodies. [file peerj-07-7406-s003.pdf]

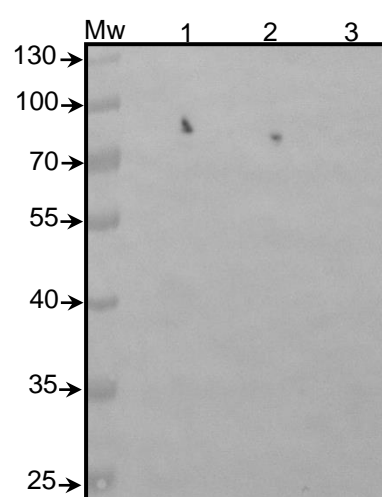

Supplement: Figure S4 — The proteins corresponding to spot 1 (lane 1; labeled “spot 1 in Fig. 4), spot 2 (lane 2; labeled “spot 2” in Fig. 4), and an adjacent major spot (lane 3; labeled “M” in Fig. 4) as negative control, excised from the two-dimensional gel of proteins from Symbiodinium KB8 cultures previously incubated under 12 h of darkness were analyzed by western blot with anti-pThr antibodies. Only the lanes corresponding to spot 1 (lane 1) and spot 2 (lane 2) showed the presence of SmicHSP75. [file peerj-07-7406-s004.pdf]
